# Supplementary material for: Mitigating belief projection in explainable artificial intelligence via Bayesian teaching
Source: Sci Rep. 2021 May 10;11:9863. doi: 10.1038/s41598-021-89267-4 (PMC8110978; doi:10.1038/s41598-021-89267-4)
Supplement: Supplementary file 4 — Supplementary Information. [file 41598_2021_89267_MOESM4_ESM.pdf]

Supplementary information: Mitigating belief projection in explainable artificial intelligence via Bayesian Teaching

Scott Cheng-Hsin Yang,[1†*], Wai Keen Vong,[2†] Ravi B. Sojitra,[3†], Tomas Folke[1], Patrick Shafto[1]

[1]Department of Mathematics and Computer Science, Rutgers University
101 Warren Street, Newark, NJ 07102, USA
[2]Center for Data Science, New York University
60 5th Ave, New York, NY 10011, USA
[3]Department of Management Science and Engineering, Stanford University
[†]Equal contribution.
[*]To whom correspondence should be addressed; E-mail: scott.cheng.hsin.yang@gmail.com.

Supplementary Table T1

ImageNet categories used in the experiment. The 83 categories and their corresponding category accuracy are given in this table. The accuracy scores are computed on the test set of *ImageNet 1K* over the 100 selected categories described in Methods. See separate csv file.

Supplementary Table T2

The table lists all 167 unique pairs of categories used in the experiment along with each pair's familiarity score. See separate csv file.

Supplementary Tables T3

Coefficient tables for the 15 regression models reported in the main text. See separate excel file.

Supplementary Discussion D1: Participants prefer helpful examples

Methods

To test the subjective preference for helpful versus unhelpful or random examples, we use a different task. In a trial of this task, we presented a target image, its category, and two sets of example pairs for that category. The participants were asked to select which pair they think influenced the AI's classification more. Note that this experiment is different from the 2AFC experiment described previously in that there is only one category and the decision is between teaching sets.

Helpful examples are chosen to be the teaching examples for the target category in trials where $f_L(\cdot) > 0.8$. Likewise, unhelpful examples are chosen to be the examples for the target category in trials where $f_L(\cdot) < 0.2$. On average, these examples are expected to be helpful or detrimental regardless of what the other category is; thus, they can be approximated as examples that aim to maximize or minimize the marginal teaching probability. We extracted 67 target images that have both helpful examples and unhelpful examples. Given a target image's category, random examples are simply random samples from the training images in *ImageNet 1K* that are not the target image or the helpful examples.

80 participants (25 male, 54 female, 1 other) were recruited from Amazon Mechanical Turk and paid $1.00 for completing the experiment, which took roughly 5 minutes to complete. The participants were randomly assigned to one of the two conditions (helpful-vs-unhelpful and helpful-vs-random) with 40 in each condition. The mean age of participants was 36.7 years (SD = 10.5), ranging from 16 to 68 years. 6 participants were excluded from analysis for completing the experiment too quickly (less than one second per trial), resulting in a final sample of 74 participants.

The study protocol was approved by Rutgers University IRB. All research was performed in accordance with the approved study protocol. An IRB-approved consent page was displayed before the experiment. Informed consent was obtained from all participants. The experiment began after the participants gave consent.

Results

We wanted to evaluate whether participants preferred informative to uninformative and misleading examples. To test this, we ran a second study where participants chose between helpful examples versus random examples (n=37) or versus unheplful examples (n=37). The helpfulness of the examples is determined by Bayesian Teaching. The helpful examples are chosen to best represent the target category by maximizing the marginal teaching probability; random examples are uniformly randomly sampled from the target category; and unhelpful examples are chosen to mislead the learner to infer any other category by minimizing the marginal teaching probability. The marginal teaching probability is the probability that a set of examples will lead the explainee model to infer the target category compared to any other category in a 2AFC task (see Methods for more details).

Participants showed a small but reliable preference for helpful relative to random examples (53.05% [95% CI = 51.08% - 55.01%], z=3.03, p = .002) and a substantial preference for helpful versus to unhelpful examples (64.14% [95% CI = 61.68% - 66.59%], z=10.95, $p < .0001$). These two conditions were reliably different ($\chi 2 = 36.94$, $p < .0001$), implying that the Bayesian Teacher

is not only capable of selecting helpful examples, but can also select examples that are actively confusing (see Figure D1-1). As this pattern of preferences matches our predictions as stated in the introduction, a natural next steps is to evaluate whether these preferences are particularly pronounced for unfamiliar examples, as hypothesised. We found that participants were more likely to prefer helpful examples when the choice categories were unfamiliar to them (β= -0.57(0.08), z = -7.02, p $<$ .0001), irrespective of whether helpful examples were contrasted with random or unhelpful examples.

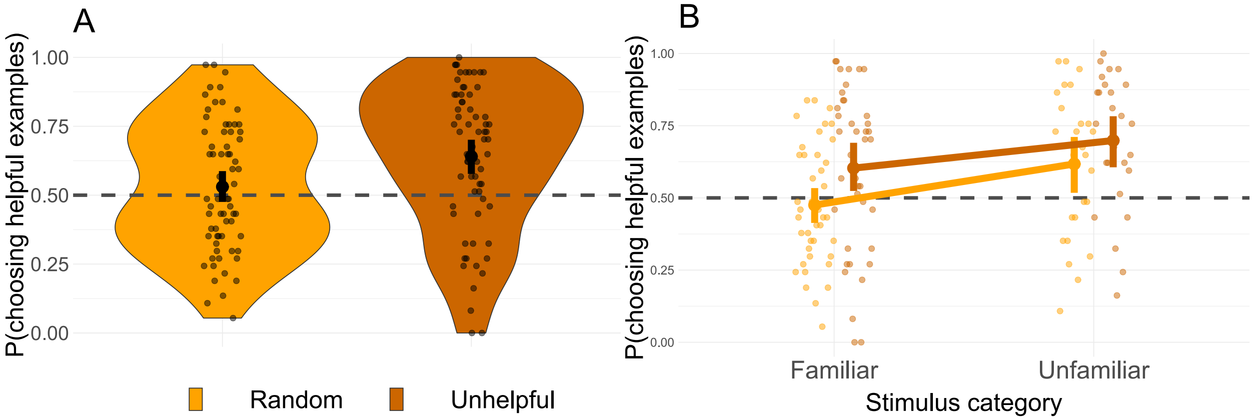

Figure D1-1: Helpful examples are preferred to unhelpful and random examples, especially for unfamiliar categories. **(A)**. The probability that a participant chose helpful examples over random (37 participants; 2479 observations), or unhelpful examples (37 participants; 2479 observations) respectively. **(B)**. The less familiar participants are with the stimulus categories, the more they prefer helpful examples. Familiarity ratings were continuous in the analyses reported in the main text, but are dichotomized here for visual clarity. Each transparent point represents the average probability across participants for one specific stimulus pair. Solid points represent the mean across all stimulus pairs. Error bars signify 95% bootstrapped confidence intervals. This figure was created using the ggplot2 package (v. 3.3.2) [1] in R (v. 4.0.3) [2].

Supplementary Discussion D2: Analysing MAP conditions separately

In the main text we combined the two MAP] conditions in our analyses. To show that this decision did not meaningfully impact our conclusions we repeat the same analyses with [JET] and [BLUR] as separate predictors here. We ran hierarchical logistic regressions on the complete dataset predicting the fidelity between the participant predictions of the classifications of the AI model and its actual classifications based the explanatory interventions ([SPECIFIC-LABELS] vs [GENERIC-LABELS], [BLUR] vs [JET] vs [NO MAP], and [EXAMPLES] vs [NO EXAMPLES], while controlling for category accuracy and familiarity ratings.

[BLUR] improves fidelity when the AI classifier is wrong (β= 0.43(0.03), z = 12.27, $p < .0001$), as do [JET] (β= 0.43(0.03), z = 12.34, $p < .0001$). However, the saliency maps reduce fidelity (to a lesser extent) when the AI classifier is correct, both for [BLUR] (β= -0.49(0.08), z = -6.06, $p < .0001$) and for [JET] (β= -0.62(0.08), z = -7.76, $p < .0001$), see Figure D2-1. In both cases, the saliency maps reduced the first order-accuracy of the participants. [BLUR] AI correct: β= -0.49(0.08), z = -6.05, $p < .0001$, AI wrong: β= -0.43(0.03), z = -12.27, $p < .0001$; [JET] AI correct: β= -0.62(0.08), z = -7.76, $p < .0001$, AI wrong: β= -0.43(0.03), z = -12.34, $p < .0001$. This reduction in first-order accuracy means that participants were less likely to believe that the AI judgements matched the ground truth of the image. This in turn implies that the saliency maps encourage participants to consider that the AI might be mistaken. This interpretation assumes that participants know the ground truth for most of the trials, which seems plausible given typical human classification accuracy on the ImageNet dataset [3].

The familiarity ratings capture the ease of the discrimination task in that they are higher for trials involving categories that humans are familiar with. We can use these ratings to further explore whether participants project their own beliefs onto the AI. Specifically, if humans use their first-order classifications to model the AI, familiarity should positively correlate with fidelity when the AI is correct, but negatively correlate with fidelity when the AI is wrong. This is indeed what we find: participants are more likely to accurately predict AI classifications when they are familiar with the item categories and the AI is correct (β= 1.10(0.04), z = 29.28, $p < .0001$), but they are less likely to correctly predict AI errors (β= -0.92(0.02), z = -42.82, $p < .0001$). In other words, participants are more likely to assume that the AI gets it right for trials that they themselves find easy.

Previously we showed that saliency maps improved prediction accuracy on trials when the AI was wrong. We suggested that this might be explained by saliency maps helping participants distinguish between their first-order judgements of the ground truth and their fidelity when predicting the model classification. This can be evaluated directly by testing whether the impact of the familiarity ratings on classification accuracy are attenuated by the saliency maps (see Figure D2-1). In other words, if participants are more likely to predict that the AI is correct on trials that they themselves find easy, and the saliency maps work by helping people realise that the AI use different decision-processes, the saliency maps should make participants more willing to consider that the AI is wrong for trials they themselves find easy. This is what we find, see Figure D2-1). Specifically, the presence of [BLUR] maps reduces the positive impact of familiarity on fidelity when the AI is correct (β= -0.61(0.09), z = -6.67, $p < .0001$) and the same is true for [JET] maps (β= -0.44(0.09), z = -4.87, $p < .0001$). Conversely, saliency maps reduce the negative impact of

familiarity on fidelity when the AI is wrong, for both [BLUR] (β= 0.74(0.05), z = 13.95, $p < .0001$) and [JET] (β= 0.67(0.05), z = 12.73, $p < .0001$). Collectively these results suggest that the presence of saliency maps help participants model the AI as an agent with distinct beliefs that may conflict with their own.

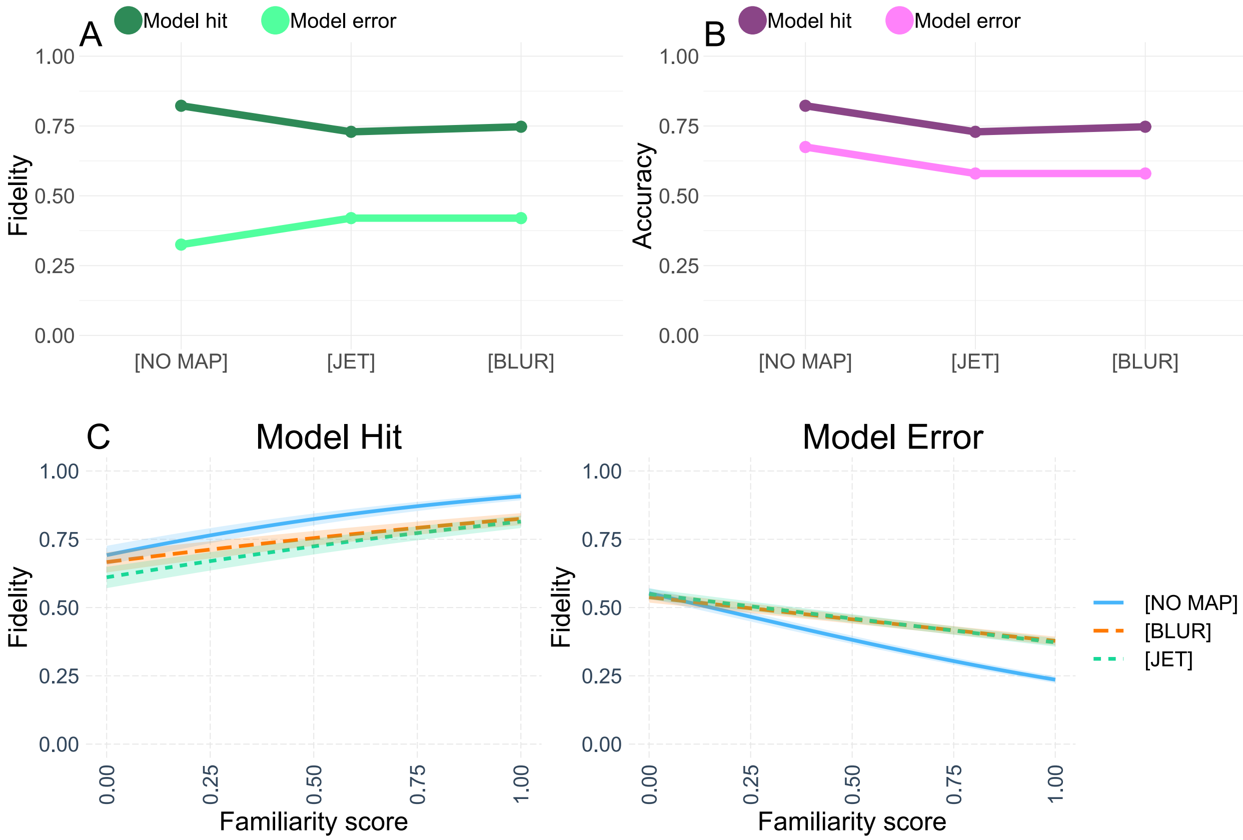

Figure D2-1: Saliency maps improve human fidelity when identifying model errors, and reduce fidelity when identifying model hits, irrespective of the presentation format of the saliency maps. All subplots are based on the entire data set, comparing [JET] and [BLUR] and [NO MAP] conditions (631 participants; 94,582 observations). **(A)**. The saliency maps improve fidelity for trials when the AI is wrong but reduce fidelity when the AI is correct. **(B)**. The saliency maps make people less likely to classify the target image to align with the ground truth, independent of AI accuracy. Together, A & B imply that the saliency maps help people to consider that the AI might make mistakes. **(C)** Saliency maps decrease the impact of familiarity on participant judgements. For model hits this leads to decreased fidelity, whereas for model errors it leads to improved fidelity. This pattern provides further evidence that the saliency maps work by shifting participants away from using their first-order judgments to model the AI classifications. Collectively these figures suggest that [JET] and [BLUR] have very similar impacts on participant judgements. Errorbars represent 95% bootstrapped confidence intervals. All point estimates have confidence intervals, though some are too narrow to see clearly. Shaded areas represent analytic 95% confidence intervals. This figure was created using the ggplot2 package (v. 3.3.2) [1] in R (v. 4.0.3) [2].

Supplementary Figure F1: The relationship between category accuracy and participant fidelity in the control condition

Focusing exclusively on the control trials, we see that category accuracy is positively associated with human fidelity when the AI is wrong (β= 0.81(0.11), z = 6.95, $p < .0001$), but even more so when the AI is correct (β= 0.92(0.23), z = 3.96, $p < .0001$).

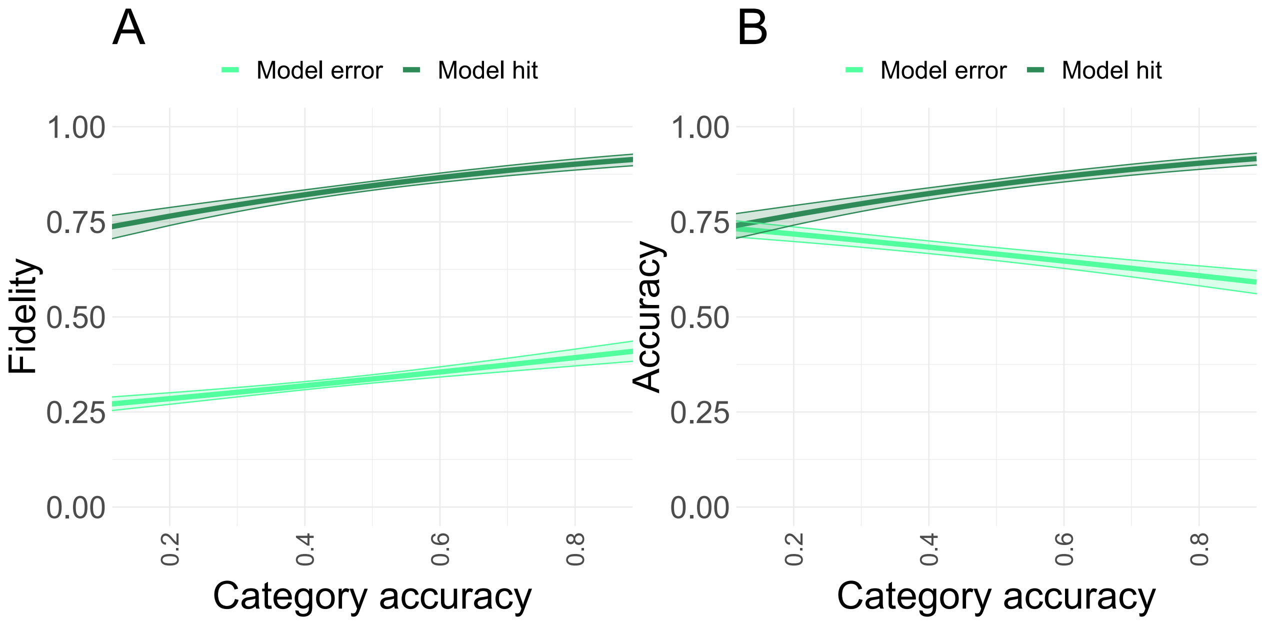

Figure F1: category accuracy versus participant fidelity in the control condition. **(A)** category accuracy is positively associated with human fidelity during the control condition both for trials when the AI is correct and when the AI is wrong. However, the base rate human fidelity is much higher when the AI is correct. **(B)** The probability that the participant judgement corresponds to the ground truth is positively associated with category accuracy when the model is correct, but negatively associated with category accuracy when the model is wrong. Both subplots are based on the control trials only (76 participants; 11,394 observations). Shaded areas represent analytic 95% confidence intervals. This figure was created using the ggplot2 package (v. 3.3.2) [1] in R (v. 4.0.3) [2].

References

[1] Hadley Wickham. *ggplot2: Elegant Graphics for Data Analysis*. Springer-Verlag New York, 2016.

[2] R Core Team. *R: A Language and Environment for Statistical Computing*. R Foundation for Statistical Computing, Vienna, Austria, 2020.

[3] Olga Russakovsky, Jia Deng, Hao Su, Jonathan Krause, Sanjeev Satheesh, Sean Ma, Zhiheng Huang, Andrej Karpathy, Aditya Khosla, Michael Bernstein, Alexander C. Berg, and Li Fei-Fei. ImageNet Large Scale Visual Recognition Challenge. *International Journal of Computer Vision (IJCV)*, 115(3):211–252, 2015.
